# Superiority of Organic Calcium Fertilizers over Inorganic Counterparts in Enhancing Fruit Firmness and Quality of Blue Honeysuckle (*Lonicera caerulea* L.)

**DOI:** 10.3390/foods15111856

**Published:** 2026-05-24

**Authors:** Yuxi Chen, Wei Li, Xuefei Ji, Haihui She, Pengke Yan, Yue Xiao, Hao Sun, Junwei Huo

**Affiliations:** 1 College of Horticulture and Landscape Architecture, Northeast Agricultural University, Harbin 150038, China; 2 College of Resources and Environment, Northeast Agricultural University, Harbin 150030, China; 3 Harbin Ecology and Agriculture Meteorological Center, Harbin 150028, China; 4 National Key Laboratory of Smart Farm Technologies and Systems, Northeast Agricultural University, Harbin 150030, China

**Keywords:** blue honeysuckle, calcium fertilizer, fruit firmness, postharvest quality

## Abstract

Blue honeysuckle is an emerging small berry with high nutritional value; However, the soft texture of its fresh fruit leads to poor storability and transportability, severely limiting its economic returns. Calcium is a key element in maintaining the structural integrity of plant cell walls and membranes, and plays a decisive role in fruit firmness and postharvest quality. To elucidate the effects of different calcium fertilizers on the fruit firmness, yield, and fruit quality of blue honeysuckle, a two-year field experiment was conducted using foliar application of 2 L of calcium fertilizer (CaC, CaA, CaL, SAC and AAC) at a concentration of 1 mg/L, with the control receiving an equal volume of water. The results showed that sugar alcohol calcium had the best effect on fruit firmness, fresh weight, yield, solid-acid ratio, total anthocyanins, and ascorbic acid, significantly improving yield and quality. Amino acid calcium was most effective in increasing fruit volume and fruit number per plant. Calcium acetate and calcium lactate showed balanced improvements across indicators. Calcium chloride significantly enhanced fruit shape index, total phenols, and total flavonoids. These findings elucidate the specific effects of various treatments on key fruit traits, providing a theoretical basis for precision cultivation and quality regulation, thereby facilitating the standardization and high-quality development of the industry.

## 1. Introduction

Blue honeysuckle, scientifically named *Lonicera caerulea* L., is a perennial shrubby fruit tree belonging to the genus *Lonicera* of the family Caprifoliaceae. Its fruit is a small berry, mainly distributed in the Greater Khingan Mountains, Lesser Khingan Mountains, and Changbai Mountains in China [1,2]. Blue honeysuckle is primarily cultivated, with nearly 6000 hectares planted in northeastern China, yielding 4–13 tons per hectare. It has a fruit-bearing period of about 15 years, tolerates severe cold down to −50 °C, and its fruits can be eaten fresh or processed. Blue honeysuckle is rich in bioactive substances such as anthocyanins, ascorbic acid, flavonoids, and phenolic acids, and has high economic and nutritional value [3]. It has been developed as a fresh-eating cultivar [4]. However, blue honeysuckle is prone to over-softening during harvesting. The pulp of over-softened blue honeysuckle loses its taste and flavor, and is more susceptible to rot and deterioration. This not only limits the storage and transportation of blue honeysuckle, but also increases the difficulty of postharvest handling [5]. Previous studies have shown that foliar application of calcium fertilizer is one of a few important measures that alleviate the softening of some berry fruits, such as grapes and blackberries [6,7]. However, it remains unclear whether calcium fertilizer application can increase the fruit firmness of blue honeysuckle, and how different types of calcium fertilizers affect the fruit quality and postharvest preservation effect of blue honeysuckle.

Calcium is an essential nutrient for plants and plays a key role in maintaining the structure and function of cell walls and cell membranes [8]. Calcium deficiency leads to loose cell wall structures and reduced pectin cross-linking, which results in fruit softening, poor storage and transport resistance, and high susceptibility to postharvest rot [9]. For instance, foliar application of calcium fertilizer during grape growth can significantly increase the soluble solid content of ripe fruits and reduce the fruit cracking rate [10]. Secondly, calcium is involved in cellular signal transduction; calcium deficiency aggravates the physiological metabolism of postharvest fruits and accelerates quality deterioration [9]. Foliar calcium application can effectively alleviate the rapid rot of fruits within 3–4 days after harvest, such as red currants [11]. However, due to the weak mobility of calcium in plants, it is vividly referred to as “lazy calcium”. Therefore, foliar spraying has become an important calcium supplementation method, as it can directly act on the fruit surface or functional leaves [12]. Thus, foliar application of calcium fertilizer is of positive significance for fruit growth and development, firmness enhancement, and postharvest storage. Erbaş and Koyuncu [13] found that CaCl_2_ treatment significantly inhibited the respiration rate of cherries and increased fruit firmness. Ma et al. [14] demonstrated that the combination of foliar and fruit cluster spraying with rhizosphere irrigation of sugar alcohol calcium increased the pulp firmness of Jufeng grapes by 19.28%, significantly enhanced the contents of total phenols and anthocyanins, and reduced the fruit cracking rate. Foliar calcium spraying is one of the most commonly used calcium supplementation technologies, which can quickly meet the calcium demand for fruit growth and development through epidermal cells, and has become an important means of supplementing calcium nutrition in agricultural production [15,16]. Studies have shown that exogenous calcium treatment can alter the activity of cell wall-degrading enzymes, enhance cell wall stability, improve the firmness and storage performance of blue honeysuckle, and affect the accumulation of sugars and acids as well as the synthesis of secondary metabolites by regulating related metabolic pathways, ultimately improving its commercial and nutritional quality.

Common foliar-applied calcium fertilizers in production can be roughly classified into inorganic calcium fertilizers and organic calcium fertilizers. Inorganic calcium fertilizers, such as calcium chloride, exist in the form of free Ca^2+^. Their advantages include low cost and good water solubility; however, Ca^2+^ is easily fixed by soil. During spraying, it tends to bind to the negative charge sites on the leaf surface, resulting in poor mobility, difficulty in direct absorption by fruits, and potential leaf burn at high concentrations [8,17]. In contrast, organic calcium fertilizers (e.g., amino acid calcium, sugar alcohol calcium) chelate or complex Ca^2+^ through organic ligands, thereby improving calcium solubility, stability and bioavailability in plants. This structure protects calcium ions, enabling them to more easily penetrate the leaf cuticle and be transported in the phloem of plants. Thus, calcium can be more efficiently delivered to fruit parts with weak transpiration, significantly improving the utilization efficiency and bioactivity of calcium to facilitate absorption, and may enhance the physical durability of fruits [18,19]. Currently, research on the quality regulation of common fruit trees such as grapes, apples, and kiwifruits by calcium fertilizers is relatively in-depth, but the differences in the effects of calcium fertilizers with different formulations on the quality of blue honeysuckle remain unclear. As a characteristic berry with high anthocyanin content, the correlation between the mechanisms of firmness enhancement and quality formation of blue honeysuckle and calcium metabolism, as well as the suitable type of calcium fertilizer and spraying scheme, all require systematic investigation. Based on this, this study takes blue honeysuckle as the test material to explore: 1. What are the differences in the effects of different calcium fertilizers on improving fruit firmness? 2. How do different calcium fertilizers affect changes in fruit nutrients? Different forms of calcium fertilizers were used for foliar spraying treatment, and key indicators such as fruit quality and functional components were determined to clarify the application effects, advantages and disadvantages of different calcium fertilizers, so as to provide a theoretical basis and technical support for the rational application of calcium fertilizers in the high-quality and high-yield cultivation of blue honeysuckle.

## 2. Materials and Methods

### 2.1. Field Experiment

**Figure 1:**
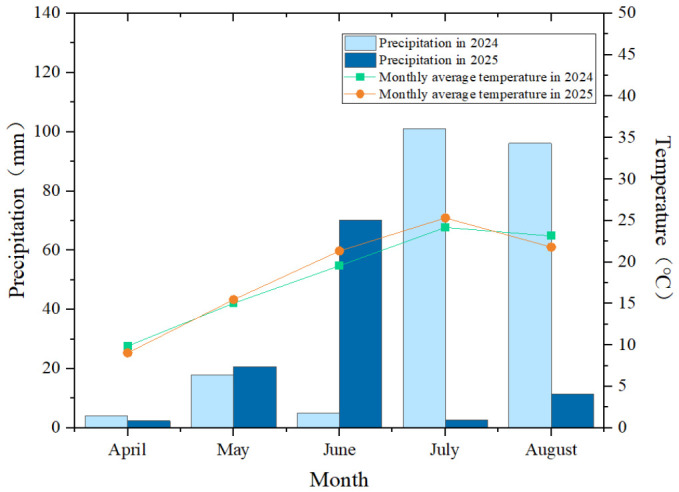
Precipitation and temperature in April–August 2024 and 2025.

The experimental site was selected at the Xiangyang Base of Northeast Agricultural University in Xiangfang District, Harbin City, Heilongjiang Province (126°93′ E, 45°76′ N), which possesses a temperate continental monsoon climate and belongs to the first accumulated temperate zone in Heilongjiang Province. In 2024, the rainfall in this area was mainly concentrated in July and August, with low rainfall in June (Figure 1). The rainfall from April to August was 224.1 mm, the average annual rainfall was 530 mm, the average annual temperature of the experimental site was 4.3 °C, the annual sunshine hours were about 2641~2732 h, the effective accumulated temperature (≥10 °C) was about 2300~2500 °C·d, and the frost-free period was about 110~125 days. In 2025, the rainfall was concentrated in June, and the rainfall in July and August was less (Figure 1). The rainfall from April to August was 107.5 mm, the average annual rainfall was about 600 mm, the frost-free period contained about 130–150 days, the average annual temperature was about 5.6 °C, the annual sunshine hours were about 2460–2786 h, and the effective accumulated temperature (≥10 °C) was about 2700–2900 °C·d. April to July sees the main stage of growth and development of blue honeysuckle, and the climate comparison in two years found that the rainfall in 2024 was mainly concentrated in the harvest period, while the rainfall in 2025 was concentrated in the fruit growth period. The average monthly temperature from May to July 2024 was lower than that in 2025.

### 2.2. Experiment Design

In this study, a 5-year-old blue honeysuckle plant was selected, planted on 5 June 2019, and green branch cuttings were used. The row spacing of the plants is mostly 1 × 3.5 m, the weeding and loosening of the soil under the trees are carried out manually after the leaves fall every autumn, and the peat is covered between the rows to ensure the growth of the root system. Every year, at the beginning of spring budding (10 April) and after fruit harvest (15 July), urea (total nitrogen content ≥ 46%, particle size range: 1.18–3.35 mm, Anhui Haoyuan Chemical Group Co., Ltd., Hefei, China), diammonium phosphate (DPA, N + P_2_O_5_ ≥ 64%, Yunnan Sanhuan Sinochem Fertilizer Co., Ltd., Kunming, China), and microbial fertilizer (concentrated microbial preparation of *Bacillus subtilis*, microbial agent *Trichoderma harzianum*, effective viable count ≥ 20 billion/g, Shandong Hairuisi Marine Biotechnology Co., Ltd., Qingdao, China) were applied in a ratio of 5:2:10, and the fertilization rate per plant was 0.25 kg.

In the test for two consecutive years, the test material was the consistent growth of healthy and pest-free ‘Lan jingling’ blue honeysuckle, and the calcium fertilizers for the test included calcium chloride (calcium chloride content ≥ 96%, Tianjin Hengxing Chemical Reagent Manufacturing Co., Ltd., Tianjin, China), calcium acetate (calcium acetate content ≥ 98%, Fuchen Chemical Reagent Co., Ltd., Tianjin, China), calcium lactate (calcium lactate content ≥ 98%, Fuchen Chemical Reagent Co., Ltd.), sugar alcohol calcium (Ca ≥ 140 g/L, Brandt Co., Ltd., Springfield, IL, USA), and calcium amino acid (amino acid ≥ 100 g/L, Ca ≥ 100 g/L, Sichuan Guoguang Agrochemical Co., Ltd., Sichuan, China).

A completely randomized block design was adopted in this experiment, with a total of six treatments: control; calcium chloride (CaC); calcium acetate (CaA); calcium lactate (CaL); sugar alcohol calcium (SAC); amino acid calcium (AAC). All concentrations were set at 1.0 g/L, and distilled water was sprayed in the control group. Each treatment included five blue honeysuckle plants, totaling 30 plants. Foliar spraying was conducted at the young fruit stage, fruit expansion stage, and fruit color transition stage of the plants in 2024 and 2025. A manual pneumatic sprayer was used for spraying before 10:00 a.m., with the spraying standard being until the leaves and fruit surfaces were dripping with the solution.

### 2.3. Sampling and Measurements

In this experiment, fruits were harvested on 27 June 2024 and 18 June 2025, both at 47 days after flowering. All fruits from each individual plant were collected into harvest containers, the number of fruits was recorded, and the yield per plant was weighed using an electronic balance. After the yield measurement, 24 fruits were randomly selected for each plant for each index measurement, that is, 120 fruits were picked for each treatment and marked. The appearance quality and flavor compounds of fruits are highly susceptible to environmental influences and should be determined immediately after harvest. In contrast, indicators related to nutritional components and enzyme activities are relatively unstable and prone to oxidative degradation; therefore, graded low-temperature preservation is recommended. Storage at 4 °C significantly inhibits the activity of polyphenol oxidase (PPO), reduces the oxidation and degradation of anthocyanins, phenolics, and other compounds, and is suitable for short-term sample preservation. Ultra-low temperature freezing at −80 °C essentially terminates enzymatic reactions and microbial activity, enabling long-term stable preservation of samples and ensuring the accuracy and reliability of subsequent measurements.

Fruit morphology: After yield measurement, the weight of the fruit per fruit was measured with an electronic balance. The longitudinal and transverse diameters of the fruit were measured by vernier calipers (Deli DL91150, Deli Group Co., Ltd., Ningbo, China, precision 0.01 mm), and the fruit shape index and fruit volume were calculated according to Formulas (1) and (2) [20,21]. The fruit firmness was determined by a texture meter (Ametek Trading (Shanghai) Co., Ltd., Shanghai, China).

(1) $$\begin{array}{c} Fruit\;shape\;index=\frac{\mathrm{fruit}\;\mathrm{longitudinal}\;\mathrm{diameter}}{\mathrm{fruit}\;\mathrm{transverse}\;\mathrm{diameter}} \end{array}.$$

(2) $$\mathrm{Fruit}\;\mathrm{volume}\;({\mathrm{cm}}^{3})=\frac{\pi }{6}\times {\mathrm{transverse}\;\mathrm{diameter}}^{2}\times \mathrm{longitudinal}\;\mathrm{diameter}.$$

Fruit soluble solids and organic acids: After fruit yield measurement, the content of soluble solids (Brix°) and organic acid (%) was measured using a handheld sugar–acid meter (ATAGO PAL-BX/ACID F5, ATAGO Co., Ltd., Fukaya, Saitama, Japan), and the solid-acid ratio was calculated according to Formula (3):

(3) $$\begin{array}{c} Solid\text{-}acid\;ratio=\frac{\mathrm{soluble}\;\mathrm{solids}}{\mathrm{organic}\;\mathrm{acids}} \end{array}.$$

Total anthocyanin content in fruit was determined using the pH differential method [22]. The specific procedure was as follows: 0.5 g of blue honeysuckle sample was weighed and ground into a homogenate with 60% hydrochloric acid-ethanol solution. The homogenate was then diluted to 5 mL. After ultrasonic extraction in a water bath, a 10 mL aliquot of the homogenate was centrifuged to obtain the supernatant for analysis. An aliquot of 0.5 mL of the extract was mixed with 4.5 mL of pH 1.0 buffer. Separately, another 0.5 mL aliquot of the extract was mixed with 4.5 mL of pH 4.5 buffer. Both mixtures were placed in the dark for 60 min. The absorbance was measured at 510 nm and 700 nm. The total anthocyanin content was calculated according to Equations (4) and (5), where “MW” is the molecular weight of cyanidin-3-glucoside (449.2), “DF” is the dilution factor, “ε” (epsilon) is 26,900, and “m” is the sample mass (g):

(4) $$\begin{array}{c} A=\left(A510-A700\right)pH\;1.0-\left(A510-A700\right)\;pH\;4.5, \end{array}$$

(5) $$\begin{array}{c} Total\;anthocyanin\;content\;\left(\mathrm{mg}/g\right)=\frac{A\times \mathrm{MW}\times \mathrm{DF}\times 1000}{\varepsilon \times m} \end{array}.$$

The total phenolics content was determined using the Folin–Ciocalteu method [23]. The specific procedure was as follows: 2 g of blue honeysuckle sample was weighed and ground into a homogenate with 60% ethanol solution. The homogenate was then diluted to 50 mL and centrifuged to obtain the supernatant for analysis. An aliquot of 1 mL of the extract was transferred into a test tube, followed by sequential addition of 1 mL of Folin–Ciocalteu reagent and 3 mL of 20% sodium carbonate solution. The mixture was diluted to 25 mL with distilled water, mixed thoroughly, and allowed to stand for 15 min. Subsequently, the reaction mixture was incubated in a water bath at 25 °C for 2 h. The absorbance was measured at a wavelength of 765 nm. The total phenolics content was calculated using Formula (6), where “C” is the total phenolic content in the test solution obtained from the standard curve, “V” is the total extract volume, “n” is the dilution factor, and “m” is the sample mass:

(6) $$\begin{array}{c} Total\;phenolic\;content\;(mg/g)\;=\frac{C\times V\times n}{m\times 1000} \end{array}.$$

Total flavonoid content in fruit was determined using the aluminum nitrite-sodium nitrite method [23]. The specific procedure was as follows: 0.5 g of sample was weighed and ground into a homogenate with 60% ethanol solution. The homogenate was then diluted to 5 mL and subjected to ultrasonic extraction in a water bath for 30 min. After centrifugation, the supernatant was collected for analysis. A 3 mL aliquot of the sample solution was transferred into a test tube, and 1 mL of 5% sodium nitrite solution was added, mixed well, and allowed to stand for 6 min. Then, 1 mL of 10% aluminum nitrate solution was added, mixed well, and allowed to stand for another 6 min. Subsequently, 1 mL of 4% sodium hydroxide solution was added, mixed thoroughly, and allowed to stand for 15 min. The absorbance was measured at a wavelength of 510 nm. The total flavonoid content was calculated using Formula (7), where “C” is the content of total flavonoids in the test solution obtained from the standard curve, “V” is the total volume of the extract, “n” is the dilution factor, and “m” is the sample mass:

(7) $$\begin{array}{c} Total\;flavonoid\;content\;(mg/g)\;=\frac{C\times n\times V}{m} \end{array}.$$

Ascorbic acid content in fruit was determined using the molybdenum blue colorimetric method [24]. The specific procedure was as follows: 2 g of blue honeysuckle sample was weighed and ground into a homogenate with oxalic acid-EDTA solution. The homogenate was then diluted to 25 mL. A 1.5 mL aliquot of the homogenate was centrifuged to obtain the supernatant for analysis. A 1 mL aliquot of the supernatant was transferred into a test tube, followed by the addition of 4 mL of oxalic acid-EDTA solution, 0.5 mL of metaphosphoric acid-acetic acid solution, 1.0 mL of 5% sulfuric acid solution, and 2.0 mL of 5% ammonium molybdate solution. The mixture was diluted to 25 mL with distilled water and mixed thoroughly. The reaction mixture was then incubated in a water bath at 30 °C for 15 min. The absorbance was measured at a wavelength of 760 nm. The ascorbic acid content was calculated using Formula (8), where “C” is the vitamin C content of the sample determined from the standard curve, “Vt” is the total volume of the sample extract, “n” is the dilution factor, “Vs” is the volume of sample extract used for analysis, and “FW” is the fresh weight:

(8) $$\begin{array}{c} Ascorbic\;acid\;content\;(mg/g)\;=\frac{C\times \mathrm{Vt}\times n}{\mathrm{Vs}\times \mathrm{FW}}. \end{array}$$

### 2.4. Statistical Analysis

The normality and homogeneity of all data were verified using the Waller–Duncan test in SPSS 29.0, and data were plotted using Origin 2021. Two-way ANOVA was employed to assess the effects of calcium treatment and inter-annual variation on the yield and fruit quality of blue honeysuckle. Where interactions were significant, one-way ANOVA followed by Duncan’s multiple range test (*p* < 0.05) was used to evaluate single-factor effects. Correlation analysis further elucidated relationships among calcium treatment, inter-annuall variability, yield, and fruit quality.

Principal component analysis (PCA) based on a correlation matrix was performed to reduce dimensionality of fruit traits, and results were visualized via a biplot using Origin 2021. Finally, Origin 2021 was used to comprehensively analyze the 13 indicators of calcium treatment in two years, with the scoring rule being one point for a single grid, and then the cumulative scores of the 13 indicators were sorted to illustrate the treatment effect of different calcium fertilizers on the yield and fruit quality of blue honeysuckle.

## 3. Results

### 3.1. The Appearance Quality and Yield of Blue Honeysuckle Fruit

**Figure 2:**
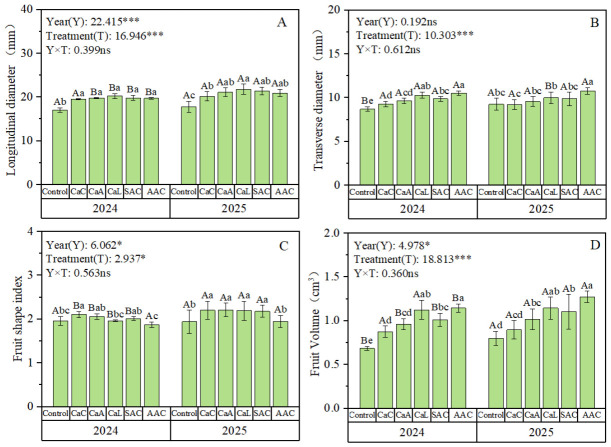
Effects of foliar application of different forms of calcium fertilizers on the appearance quality of blue honeysuckle fruits. Effects on fruit longitudinal diameter (**A**), fruit transverse diameter (**B**), fruit shape index (**C**), and fruit volume (**D**) in 2024 and 2025. Among these, the control represents spraying with water, CaC represents calcium chloride, CaA represents calcium acetate, CaL represents calcium lactate, SAC represents sugar alcohol calcium, and AAC represents amino acid calcium. T represents calcium treatment, Y represents year effect, and Y × T represents the interaction between year and calcium treatment; the values shown are F-values. Results of analysis of variance are indicated by ns (*p* > 0.05), * *p* < 0.05, and *** *p* < 0.001. Data are presented as mean ± standard deviation. Different lowercase letters indicate significant differences among different calcium form treatments (*p* < 0.05). Different uppercase letters indicate significant differences between the two years for the same calcium treatment (*p* < 0.05).

The results indicate that year and calcium treatment significantly affected fruit longitudinal diameter, fruit volume, and fruit shape index, whereas fruit transverse diameter was influenced only by calcium treatment, with no year effect. No significant year × calcium fertilizer interactions were observed for any of the four fruit morphology traits. Application of organic calcium fertilizers significantly increased fruit longitudinal diameter in 2025 compared with 2024. Across both years, all calcium fertilizers increased fruit longitudinal diameter by more than 18% relative to the control. In 2024, no differences were found among calcium fertilizers; in 2025, CaL led to significantly greater longitudinal diameter than CaC, while other organic calcium fertilizers did not differ from CaC (Figure 2A). In 2024, all calcium fertilizers increased fruit transverse diameter compared with the control, with AAC showing the greatest effect. In 2025, only AAC significantly increased transverse diameter relative to the control, and only CaL and AAC outperformed CaC (Figure 2B). For fruit shape index, in 2024, only the application of CaC resulted in significant differences in the fruit shape compared to the control group. In 2025, all treatments except CaA produced fruit shape index values that were higher than those of the control, and significantly exceeded 2024 values (Figure 2C). Fruit volume was significantly higher in 2025 than in 2024 for all calcium fertilizers except CaC and CaL. Over both years, all organic calcium fertilizers except CaA increased fruit volume by more than 40% relative to the control and >20% relative to CaC (Figure 2D).

**Figure 3:**
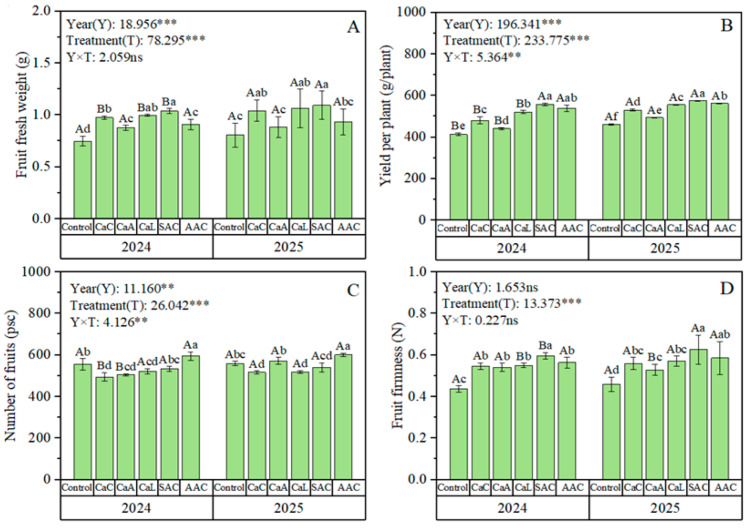
Effects of foliar application of different forms of calcium fertilizers on fruit yield per plant of blue honeysuckle fruits. Effects on fruit fresh weight (**A**), yield per plant (**B**), number of fruits (**C**), and fruit firmness (**D**) after foliar application of different calcium forms in 2024 and 2025. Among these, the control represents spraying with water, CaC represents calcium chloride, CaA represents calcium acetate, CaL represents calcium lactate, SAC represents sugar alcohol calcium, and AAC represents amino acid calcium. T represents calcium treatment, Y represents year effect, and Y × T represents the interaction between year and calcium treatment; values are F-values. Data are presented as mean ± standard deviation. Different lowercase letters indicate significant differences among different calcium form treatments (*p* < 0.05). Different uppercase letters indicate significant differences between the two years for the same calcium treatment (*p* < 0.05). Results of analysis of variance (ANOVA) are indicated by ns (*p* > 0.05), ** *p* < 0.01, and *** *p* < 0.001.

The results showed that both year and calcium treatment significantly affected fruit fresh weight, yield per plant, and fruit number, whereas fruit firmness was significantly influenced only by calcium treatment. The interaction between year and calcium treatment significantly affected yield per plant and fruit number, but not the other traits. Over the two years, fruit fresh weight in 2025 was significantly higher than in 2024 under CaC, CaL and SAC treatments, while no significant difference between years was observed under the control, CaA and AAC treatments. Across both years, SAC resulted in the highest fruit fresh weight, particularly in 2024, where it significantly outperformed all other calcium fertilizers, achieving a 7% increase compared with CaC. However, in 2025, the differences between SAC, CaC and CaL were not significant (Figure 3A). All calcium treatment significantly increased yield per plant in both years. Except for SAC and AAC, all treatments showed higher yield per plant in 2025 than in 2024. All organic calcium fertilizers, except CaA, produced greater yield increases than CaC. Among organic calcium fertilizers, SAC consistently outperformed the others, except in 2024 when its effect did not differ significantly from that of AAC. Notably, compared with conventional CaC, SAC significantly increased yield per plant by 16% and 8.3% in the two years, respectively (Figure 3B). For fruit number, the control, CaC, and CaA treatments showed significantly lower values in 2024 than in 2025, whereas no significant inter-annual differences were observed for CaL, SAC and AAC. In 2024, AAC significantly increased fruit number relative to the control, while CaL and SAC showed no significant difference from the control; CaC and CaA reduced fruit number. In 2025, AAC still significantly increased fruit number compared with the control, whereas CaC and CaL significantly decreased it; CaA and SAC had no significant effect (Figure 3C). Over the two years, all calcium fertilizers significantly enhanced fruit firmness by more than 15%. Compared with CaC, only SAC achieved the most pronounced increase, improving fruit firmness by over 9% (Figure 3D).

### 3.2. The Soluble Solids, Organic Acids and Solid-Acid Ratios of Blue Honeysuckle Fruit

**Figure 4:**
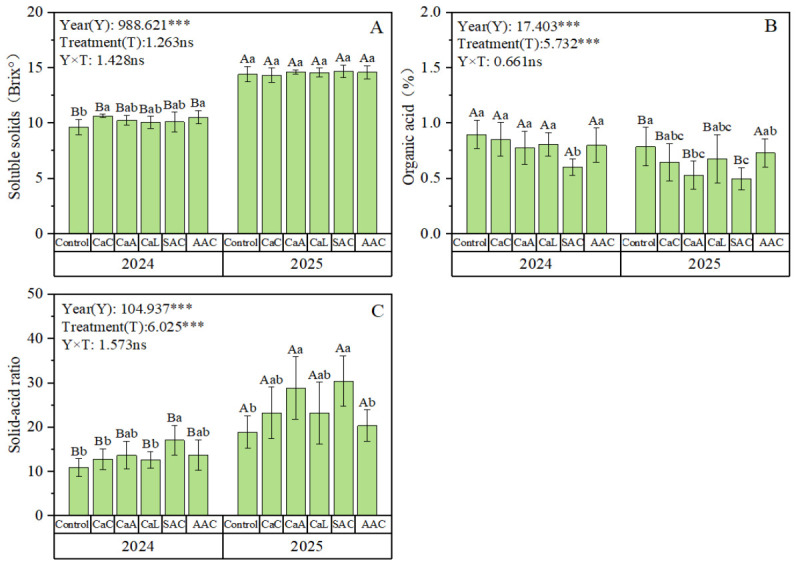
Effect of foliar application of different calcium forms on the flavor of blue honeysuckle fruit in 2024 and 2025. Effects on soluble solids content (**A**), organic acid content (**B**), and solid-acid ratio (**C**) after foliar application of different calcium forms. Among these, the control represents spraying with water, CaC represents calcium chloride, CaA represents calcium acetate, CaL represents calcium lactate, SAC represents sugar alcohol calcium, and AAC represents amino acid calcium. T represents calcium treatment, Y represents year effect, and Y × T represents the interaction between year and calcium treatment; values are F-values. Data are presented as mean ± standard deviation. Different lowercase letters indicate significant differences among different calcium form treatments (*p* < 0.05). Different uppercase letters indicate significant differences between the two years for the same calcium treatment (*p* < 0.05). Results of analysis of variance (ANOVA) are indicated by ns (*p* > 0.05), and *** *p* < 0.001.

The results showed that soluble solids of blue honeysuckle fruit were significantly affected only by year, not by calcium treatment or their interaction. In contrast, both year and calcium treatment significantly influenced organic acid and the solid-acid ratio of fruit, but their interaction was not significant. The soluble solids of blue honeysuckle fruit were significantly higher in 2025 than in 2024 across all calcium fertilizers. Only in 2024 did CaC and AAC significantly increase soluble solids compared with the control (Figure 4A). Regarding the organic acid of fruit, all treatments except AAC showed significantly lower values in 2024 than in 2025. Among calcium fertilizers, only SAC consistently reduced organic acid by more than 30% in both years, whereas CaA exerted a significant reduction only in 2025 (Figure 4B). Regarding the solid-acid ratio of fruit, all treatments exhibited significantly lower values in 2024 than in 2025. Only SAC increased the ratio by more than 55% relative to the control in both years, while CaA effectively enhanced the ratio only in 2025 (Figure 4C).

### 3.3. Nutritional Components in Blue Honeysuckle Fruits

**Figure 5:**
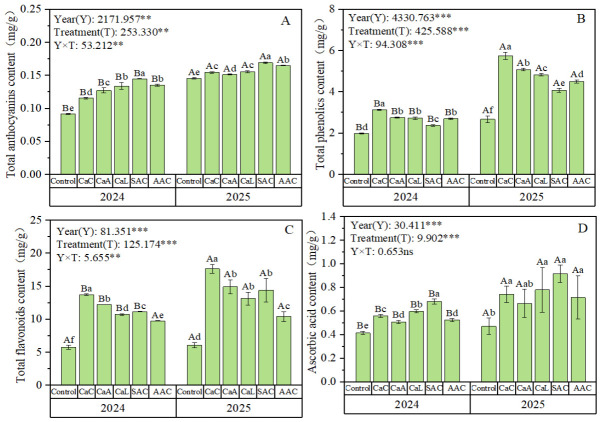
Effects of foliar application of different calcium forms on fruit quality indices of blue honeysuckle in 2024 and 2025. Effects on total anthocyanin content (**A**), total phenolics content (**B**), total flavonoid content (**C**), and ascorbic acid content (**D**) after foliar application of different calcium forms. Among these, the control represents spraying with water, CaC represents calcium chloride, CaA represents calcium acetate, CaL represents calcium lactate, SAC represents sugar alcohol calcium, and AAC represents amino acid calcium. T represents calcium treatment, Y represents year effect, and Y × T represents the interaction between year and calcium treatment; values are F-values. Data are presented as mean ± standard deviation. Different lowercase letters indicate significant differences among different calcium form treatments (*p* < 0.05). Different uppercase letters indicate significant differences between the two years for the same calcium treatment (*p* < 0.05). Results of analysis of variance (ANOVA) are indicated by ns (*p* > 0.05), ** *p* < 0.01, and *** *p* < 0.001.

The results showed that year, calcium treatment, and their interaction significantly affected total anthocyanin, total phenolics, and total flavonoid contents of blue honeysuckle fruit, whereas ascorbic acid content was significantly influenced by year and calcium treatment but not by their interaction. For total anthocyanin content, all treatments yielded significantly higher values in 2025 than in 2024. Across both years, all calcium fertilizers increased this content relative to the control. In 2024, all organic calcium fertilizers (SAC and AAC) were significantly more effective than CaC; in 2025, only SAC and AAC outperformed CaC. In both years, SAC resulted in the highest total anthocyanin content among all calcium fertilizers (Figure 5A). Total phenolics content was significantly higher in 2025 than in 2024. Across both years, all calcium fertilizers significantly increased total phenolics content compared with the control. However, contrary to the pattern observed for anthocyanins, organic calcium fertilizers enhanced total phenolics to a significantly lesser extent than CaC, with SAC showing the smallest increase (Figure 5B). For total flavonoid content, all treatments except the control and AAC showed significantly higher values in 2025 than in 2024. Similar to total phenolics, CaC was more effective than organic calcium fertilizers in promoting total flavonoid content, and AAC exhibited the smallest enhancement among organic treatments (Figure 5C). Ascorbic acid content was significantly higher in 2025 than in 2024 across all treatments. In 2024, SAC was the most effective promoter of ascorbic acid content, whereas in 2025, no significant differences were observed among calcium fertilizers (Figure 5D).

### 3.4. Comprehensive Evaluation and Principal Component Analysis of Two-Year Calcium Treatment

**Figure 6:**
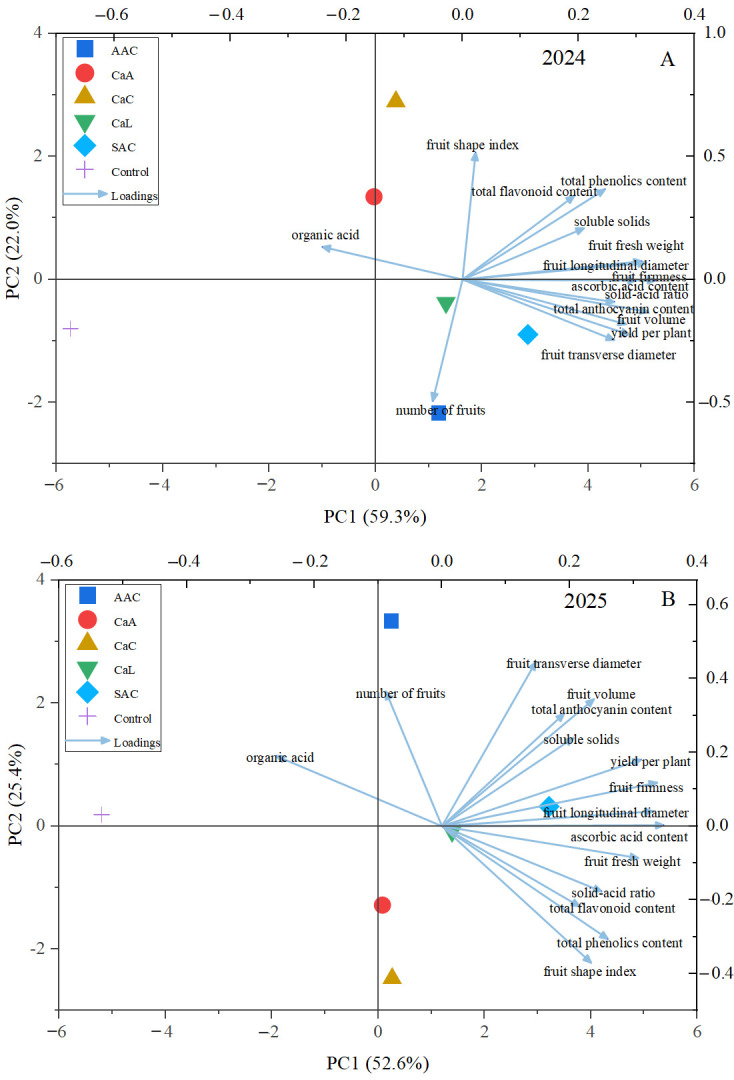
Principal component analysis of fruit traits in 2024 and 2025. Figure (**A**) shows the principal component analysis of fruit traits for 2024, and Figure (**B**) shows the principal component analysis of fruit traits for 2025.Among these, the control represents spraying with water, CaC represents calcium chloride, CaA represents calcium acetate, CaL represents calcium lactate, SAC represents sugar alcohol calcium, and AAC represents amino acid calcium.

The results showed that in 2024, principal component analysis (PCA) showed that PC1 and PC2 explained 59.3% and 22.0% of the variance, respectively, with a cumulative contribution of 81.3% (Figure 6A). The control and factor loadings (loading) were near the origin, indicating that their traits were close to the average levels. CaA, CaC, CaL, and SAC were distributed on the positive side of PC1, showing positive correlations with yield per plant, fruit volume, single fruit weight, and fruit firmness, thus representing a high-yield, large-fruit type. SAC and CaL were located on the negative side of PC2, positively correlated with the solid-acid ratio and soluble solids content, indicating superior flavor quality. AAC was located on the positive side of PC2 and was positively correlated with antioxidant components such as ascorbic acid, anthocyanins, total phenolics, and total flavonoids. In 2025, PCA results (Figure 6B) showed that PC1 and PC2 explained 52.6% and 25.4% of the variance, respectively, with a cumulative contribution of 78.0%. AAC and SAC were positioned on the positive side of PC1 and were positively correlated with all measured traits, demonstrating the best overall performance. In contrast, CaA, CaC, CaL, and the control exhibited consistently low values for all traits, with no significant differences among them. Therefore, the two-year PCA indicates that AAC exhibits the best comprehensive performance in terms of fruit yield, quality, and antioxidant components.

**Figure 7:**
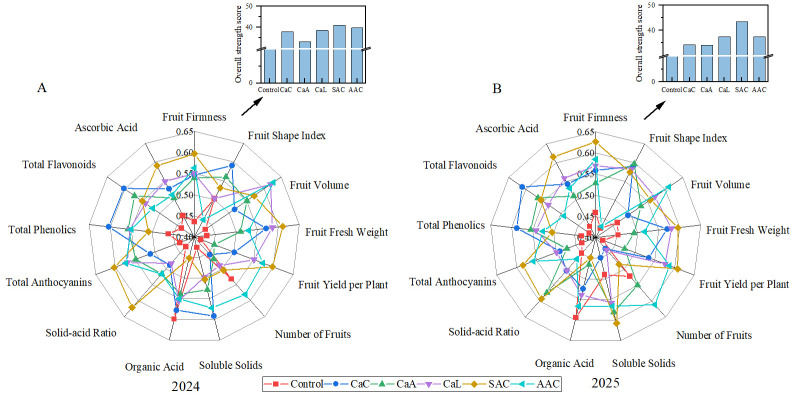
Radar chart and comprehensive score of various indices in blue honeysuckle under different calcium fertilizers in two years. Figure (**A**) shows the 2024 calcium treatment radar chart, and Figure (**B**) shows the 2025 calcium treatment radar chart.Among these, the control represents spraying with water, CaC represents calcium chloride, CaA represents calcium acetate, CaL represents calcium lactate, SAC represents sugar alcohol calcium, and AAC represents amino acid calcium. Obvious differences were observed in the polygon characteristics of radar charts among all 13 evaluation objects in 2024 and 2025. The indices in the radar chart were arranged clockwise as follows: fruit firmness, fruit shape index, fruit volume, fruit fresh weight, yield per plant, number of fruits, soluble solids, organic acid, solid-acid ratio, total anthocyanin, total phenolics, total flavonoid, and ascorbic acid. The bar chart shows the comprehensive evaluation scores derived from the radar chart analysis.

Compared with the control, different calcium fertilizers showed differences in yield traits such as yield per plant and fruit weight, and some treatments responded to total anthocyanins, total phenols, total flavonoids, and ascorbic acid functional components. The trait radar contours in 2024 and 2025 are similar, indicating that the impact of calcium treatment on fruit traits is stable, but the trait scores of individual calcium fertilizers fluctuate over two years (e.g., CaC, CaA), which may be related to inter-annual differences in environmental factors. The radar area of calcium treatment was larger than that of CK, indicating that calcium fertilizer spraying could synergistically improve the comprehensive performance of fruit yield and quality traits, among which SAC treatment performed relatively prominently in the dimension of multiple traits, such as firmness, fresh fruit weight, fruit yield per plant, organic acid, solid-acid ratio, total anthocyanins, and ascorbic acid. SAC treatment was followed by AAC, which had the best effect on fruit volume and number of fruits; CaL ranked third in the multi-trait dimension for two years, and all indicators developed in a balanced manner (Figure 7).

## 4. Discussion

### 4.1. Sugar Alcohol Calcium Fertilizer Is More Advantageous in Enhancing the Fruit Shape, Yield and Firmness of Blue Honeysuckle

Fruit shape, yield and firmness are core indicators for evaluating the economic value of fruits. This experiment found that foliar calcium application significantly affected blue honeysuckle fruit morphology, with notable inter-annual variation. Calcium treatments generally increased fruit longitudinal diameter, transverse diameter, shape index (Figures S11 and S12), and volume, consistent with the mechanism by which exogenous calcium enhances cell wall extensibility and plasticity, thereby promoting both transverse and longitudinal growth [25]. Although the year effect significantly influenced only longitudinal diameter, volume, and shape index, no year-by-treatment interaction was observed, indicating that calcium’s promoting effects remained consistent across years, while climatic factors may indirectly affect fruit shape and size via longitudinal growth and expansion. Treatment differences were more pronounced in 2025 (2 May to June 18) than in 2024 (12 May to June 27), likely due to more favorable temperature and precipitation during the fruit growth period, which amplified the treatment effects (Figure 1). Regarding volume, all organic calcium treatments except CaC and CaL showed significantly higher values in 2025 than in 2024. Except for CaA, all organic calcium treatments increased the volume by >40% relative to the control and by >20% relative to CaC, aligning with Ma et al.’s [14] study on grapes. We conclude that exogenous calcium effectively improves fruit morphology, particularly by increasing longitudinal and transverse diameters and volume, and modulating shape index. However, efficacy is influenced by inter-annual climate conditions, though consistently superior to the control across years. Compared with conventional CaC, most organic calcium treatments, especially AAC and CaL, exhibited greater advantages in enhancing volume and optimizing shape. At the same time, the molecular structures, absorption efficiencies, and interaction mechanisms with cell wall components of different organic calcium compounds require further investigation to explain the suboptimal performance of certain treatments, such as CaA, and their inter-annual variability.

This experiment found that inter-annual environmental conditions (e.g., temperature and water supply) may influence yield-related traits by modulating the efficiency of calcium uptake and utilization (Figures S9 and S10), whereas fruit hardness depends more on the direct physiological functions of calcium and is less affected by climatic fluctuations. Single fruit weight was significantly higher in 2025 than in 2024, and this inter-annual difference varied among calcium treatments. Notably, SAC significantly increased single fruit weight in 2024. However, this experiment found no significant differences from other treatments in 2025, suggesting that SAC more effectively alleviated the suppression of single fruit weight under relatively unfavorable conditions. These results are consistent with those of Smith [26] and Wu et al. [27]. Certain calcium sources amplified these environmental effects by influencing calcium signaling and cell wall assembly during cell division and expansion [28,29]. Fruit number exhibited a more complex response pattern, likely related to inter-annual differences during flower bud differentiation and the young fruit stage. AAC consistently increased fruit number in both years, indicating that amino-acid-chelated calcium promotes fruit set or reduces fruit drop [30]. In contrast, SAC did not significantly increase fruit number but achieved the highest overall yield through increased single fruit weight, implying a “large-fruit strategy” rather than a “multi-fruit strategy” [14,31]. Moreover, all calcium treatments increased fruit hardness in both years. Compared with CaC, only SAC achieved the most significant increase in hardness (Figure S10, under the treatment of SAC, the calcium content in the fruits was the highest.). This advantage may reflect that sugar-alcohol-chelated calcium is more effectively absorbed by the fruit epidermis and transported to the flesh cell wall, allowing calcium ions to bind more thoroughly with pectin to form calcium bridges, thereby more strongly inhibiting the activity of cell wall-degrading enzymes [32,33,34,35,36] (Figures S7 and S8, under the management of SAC, the activities of PG and CL were the lowest.). In conclusion, SAC exhibited stronger advantages in yield enhancement and stability under an unfavorable year (2024), whereas AAC demonstrated unique value in consistently increasing fruit number across two consecutive years.

### 4.2. Organic Calcium Fertilizer Has a More Advantageous Effect in Improving Blue Honeysuckle Fruit Quality

Fruit flavor mainly depends on total soluble solids (TSS) content, organic acid content, and solid-acid ratio. Soluble solids and organic acids are important components of blue honeysuckle pulp and are commercially used as markers for harvest maturity. This study found that fruit soluble solids content was mainly influenced by year, whereas the effects of calcium treatment and its interaction with year were not significant. However, there are inconsistencies in the conclusions of relevant studies on the effect of foliar calcium fertilizer application on fruit soluble solids content: some studies [37,38] found that foliar calcium application increased fruit sugar content, while others [39,40] pointed out that calcium fertilizer spraying had no significant effect. Our results showed that soluble solids content was significantly increased only by CaC and AAC treatments in 2024, but not by any treatment in 2025. Except for AAC, all treatments exhibited significantly lower organic acid content in 2024 than in 2025. The effect of organic versus inorganic calcium was not consistent across the two years, indicating that the stability of calcium fertilizer efficacy is regulated by year. Inter-annual climatic factors may dominate the accumulation of soluble solids and the metabolism of organic acids, with calcium treatment effects becoming apparent only under specific year-dependent conditions [41]. Regarding calcium treatments, SAC consistently reduced organic acid content by more than 30% in both years, whereas the effects of other calcium treatments were year dependent. Mechanistically, the sustained and highly effective reduction in organic acids by SAC over two years may be associated with its greater foliar absorption and conversion into endogenous calcium ions, thereby more efficiently promoting organic acid metabolism [42]. Regarding the solid-acid ratio, among all calcium treatments, SAC increased the ratio by more than 55% in both years, indicating that SAC optimizes the sugar–acid balance by reducing acidity through enhanced organic acid metabolism, thereby improving the solid-acid ratio [35,42]. Consequently, the efficacy of foliar calcium application in maintaining the sensory quality of blue honeysuckle is regulated by both year and calcium formulation. Yearly differences dominate the overall levels of soluble solids and the sugar–acid ratio, whereas calcium treatments, particularly SAC, can stably reduce organic acids and increase the sugar–acid ratio under varying annual conditions. Future research should focus on screening stable and efficient calcium formulations under different environmental conditions, while also considering the impact of inter-annual fluctuations on fruit flavor development.

Total anthocyanins, total phenolics, total flavonoids, and ascorbic acid were all significantly higher in 2025 than in 2024. In 2024, all organic calcium treatments promoted anthocyanin accumulation more effectively than CaC, whereas in 2025, only SAC and AAC outperformed CaC. A possible explanation is that in the less favorable year (2024), the efficient absorption of organic calcium became critical to compensate for environmental limitations [43]. Ascorbic acid content was independently affected by year and calcium treatment, with no interaction effect. This suggests that the regulation of ascorbic acid synthesis by calcium does not depend on annual conditions, or alternatively, that year influences only baseline metabolic activity (e.g., photosynthetic rate) without altering the relative efficacy of calcium treatments. Notably, in 2024, SAC was the most effective promoter, but no significant differences among calcium treatments were observed in 2025. This implies that in a year with milder stress or abundant resources (2025), the plant’s endogenous ascorbic acid synthesis had already reached a high level, masking any additional benefit from exogenous calcium; conversely, in the relatively unfavorable year (2024), the efficient calcium supply from SAC compensated for stress-induced ascorbic acid consumption, thereby showing a clear advantage. Overall, the significant year effect indicates that the accumulation of secondary metabolites is highly dependent on the environmental conditions during the growing season.

Interestingly, the response patterns of total anthocyanins, total phenolics, and total flavonoids to different calcium treatments were opposing. For anthocyanins, SAC and AAC were significantly more effective than CaC, with SAC being superior. In contrast, for total phenolics and total flavonoids, CaC was more effective than any organic calcium treatment; SAC resulted in the smallest increase in total phenolics, and AAC increased total flavonoids the least. SAC has higher bioavailability and phloem mobility, allowing it to be more efficiently absorbed by the fruit and thereby activating the anthocyanin biosynthesis pathway, while exerting a weaker promoting effect on the phenolic acid and flavonol branches [44,45]. Conversely, CaC has low mobility and is mainly deposited in the cell wall or apoplast, where it may stabilize cell wall structure and induce the synthesis of defense-related phenolics, thus more effectively increasing total phenolics and total flavonoids [46,47]. These findings demonstrate that the effects of different calcium sources on fruit phenolics are highly selective: organic calcium (especially SAC) is more suitable for specifically promoting anthocyanin accumulation, whereas inorganic calcium (CaC) has advantages in increasing total phenolics and total flavonoids. In addition, the significant year effect suggests that the efficacy of calcium treatments must be evaluated in the context of the climatic conditions of the specific growing season, and that the optimal application strategy should be year-specific.

### 4.3. Comprehensive Effects Evaluation of Calcium Fertilizer Forms on Blue Honeysuckle

Principal component analysis (PCA; Figure 6) revealed key information. In 2024, PC1 and PC2 accounted for 59.3% and 22.0% of the total variance, respectively (cumulative 81.3%). CaA, CaC, CaL, and SAC loaded positively on PC1, indicating high-yield and large-fruit characteristics; SAC and CaL loaded negatively on PC2, associated with superior flavor; AAC loaded positively on PC2, suggesting high antioxidant content. In 2025, PC1 and PC2 explained 52.6% and 25.4% of the variance (cumulative 78.0%). AAC and SAC were positioned on the positive side of PC1 and correlated positively with all measured traits, indicating the best overall performance. Based on the PCA results, a radar chart was constructed. The radar chart enabled a multi-dimensional comparison and analysis of 13 indicators for blue honeysuckle, revealing the distribution pattern of its comprehensive performance (Figure 6). In the two-year test, the comprehensive strength of the treatments followed the order of SAC > AAC > CaL (taking the top three), all of which had significant effects on fruit firmness. SAC also significantly improved the fruit fresh weight, yield per plant, solid-acid ratio, total anthocyanin content, and ascorbic acid content of blue honeysuckle, which has certain advantages regarding the yield increase, quality maintenance, fruit storage, transportation and nutrition of blue honeysuckle (Table S1, lower weight loss rates enhance storability). The second was AAC, which had a significant effect on increasing fruit volume and fruit number, indicating that it had a promoting effect on fruit setting and fruit expansion in blue honeysuckle. Finally, concerning CaL, the indicators develop in a balanced manner, which may be due to the small molecular weight, high activity, strong mobility, and easy absorption of CaL. The comprehensive ranking of CaC and CaA in the two-year trial changed, which may be due to the difference in the absorption efficiency of calcium fertilizer in blue honeysuckle due to the different precipitation in the two years.

Although this study provides evidence of significant differences in fruit firmness, yield, and bioactive components among treatments, the total content values obtained do not allow identification of individual phenolic or anthocyanin compounds. High-performance liquid chromatography–mass spectrometry (HPLC–MS) analysis, which can reveal treatment-specific metabolic fingerprints, would serve as a valuable complement in future research, offering deeper insights into the regulatory pathways of calcium on secondary metabolite biosynthesis. Additionally, we have included other biological activities (e.g., antioxidant capacity) in the Supplementary Materials to further advance the understanding of how calcium fertilization affects the functional quality of blue honeysuckle fruits (Figures S1–S6). Therefore, while the current conclusions remain reliable for agronomic recommendations, follow-up studies employing targeted or untargeted metabolomics based on HPLC–MS, combined with broader bioactivity assessments, are warranted to elucidate the molecular mechanisms underlying the quality improvements observed with sugar alcohol calcium and other calcium formulations.

## 5. Conclusions

Different treatment groups (e.g., CaC, CaA, etc.) had significant effects on the hardness, internal quality, and external quality of blue honeysuckle fruit, and there were year-to-year differences between 2024 and 2025. The content of various substances in the control group was lower than that in most treatment groups, while certain treatment groups (e.g., CaC and CaL in specific years) significantly increased the content of these substances. Among them, foliar application of SAC increased fruit hardness, reaching above 0.60 N, which is beneficial for fruit storage and transportation. However, in terms of increasing fruit number, SAC was slightly less effective than AAC. For increasing fruit fresh weight and yield, SAC outperformed other calcium fertilizers, achieving fruit fresh weight above 1.04 g and yield above 558.1 g, thereby achieving a dual improvement in yield and hardness at the expense of fruit number. Regarding fruit quality, SAC increased the solid-acid ratio, total anthocyanin content, and ascorbic acid content. CaC increased total phenol and total flavonoid contents, but its performance in improving the solid-acid ratio, total anthocyanin content, and fruit hardness was inferior to that of other calcium fertilizer treatments. Overall, foliar application of SAC had the best comprehensive effect.

## Data Availability

The raw data supporting the conclusions of this article will be made available by the authors on request.

## Supplementary Materials

The following supporting information can be downloaded at: https://www.mdpi.com/article/10.3390/foods15111856/s1, Figure S1: Effects of spraying different forms of calcium fertilizers on the SOD activity of blue honeysuckle fruit in 2025; Figure S2: Effects of spraying different forms of calcium fertilizers on the POD activity of blue honeysuckle fruit in 2025; Figure S3: Effects of spraying different forms of calcium fertilizers on the CAT activity of blue honeysuckle fruit in 2025; Figure S4: Effects of spraying different forms of calcium fertilizers on the PPO activity of blue honeysuckle fruit in 2025; Figure S5: Effects of spraying different forms of calcium fertilizers on the APX activity of blue honeysuckle fruit in 2025; Figure S6: Effects of spraying different forms of calcium fertilizers on the MDA content of blue honeysuckle fruit in 2025; Figure S7: Effects of spraying different forms of calcium fertilizers on the PG activity of blue honeysuckle fruit in 2025; Figure S8: Effects of spraying different forms of calcium fertilizers on the CL activity of blue honeysuckle fruit in 2025; Figure S9: Effects of spraying different forms of calcium fertilizers on the calcium content in blue honeysuckle leaves in 2025; Figure S10: Effects of spraying different forms of calcium fertilizers on the calcium content in blue honeysuckle fruits in 2025. Table S1: Weight loss rate of blue honeysuckle under room temperature and 4 °C in 2025.

## Abbreviations

The following abbreviations are used in this manuscript:

CaC	Calcium chloride
CaA	Calcium acetate
CaL	Calcium lactate
SAC	Sugar alcohol calcium
AAC	Amino acid calcium
