# Supplementary material for: Superiority of Organic Calcium Fertilizers over Inorganic Counterparts in Enhancing Fruit Firmness and Quality of Blue Honeysuckle (Lonicera caerulea L.)
Source: Foods. 2026 May 24;15(11):1856. doi: 10.3390/foods15111856 (PMC13256555; doi:10.3390/foods15111856)
Supplement: Supplementary file 1 [file foods-15-01856-s001.zip › foods-4249202-supplementary.pdf]

Supplementary Materials

Table S1. Weight loss rate of blue honeysuckle under room temperature and 4°C in 2025.

**Treatment**	**Weight loss rate (%)**	
	**Room temperature**	**4℃**
Control	63.22±0.12Aa	10.56±0.5Aa
CaC 0.5g/L	27.66±0.19Dc	10.04±0.07Aa
CaC 1.0g/L	26.94±0.06Dd	5.34±0.15Bc
CaC 2.0g/L	30.87±0.06Eb	7.77±0.17Ab
CaA 0.5g/L	49.46±0.25Ab	8.74±0.89Bb
CaA 1.0g/L	45.90±0.22Ad	7.92±0.12Abc
CaA 2.0g/L	47.34±0.22Ac	7.26±0.20Ac
CaL 0.5g/L	42.16±0.23Bb	8.19±0.54BCd
CaL 1.0g/L	29.54±0.23Cd	4.57±0.35Cc
CaL 2.0g/L	34.81±1.50Dc	5.43±0.33Bc
SAC 0.5g/L	34.56±0.84Cb	4.87±0.24Db
SAC 1.0g/L	26.42±0.13Ec	4.47±0.17Cb
SAC 2.0g/L	36.26±0.08Cb	5.09±1.41Bb
AAC 0.5g/L	27.81±0.20Dd	7.30±0.48Cb
AAC 1.0g/L	37.38±0.24Bc	4.88±0.56BCc
AAC 2.0g/L	44.21±0.65Bb	6.94±0.30Ab

In the table, the control represents spraying with water; CaC represents calcium chloride; CaA represents calcium acetate; CaL represents calcium lactate; SAC represents sugar alcohol calcium; AAC represents amino acid calcium. Data are presented as mean ± standard deviation. Different lowercase letters indicate significant differences among different concentrations of the same calcium fertilizer treatment; different uppercase letters indicate significant differences among different calcium fertilizer treatments at the same concentration ($P < 0.05$).

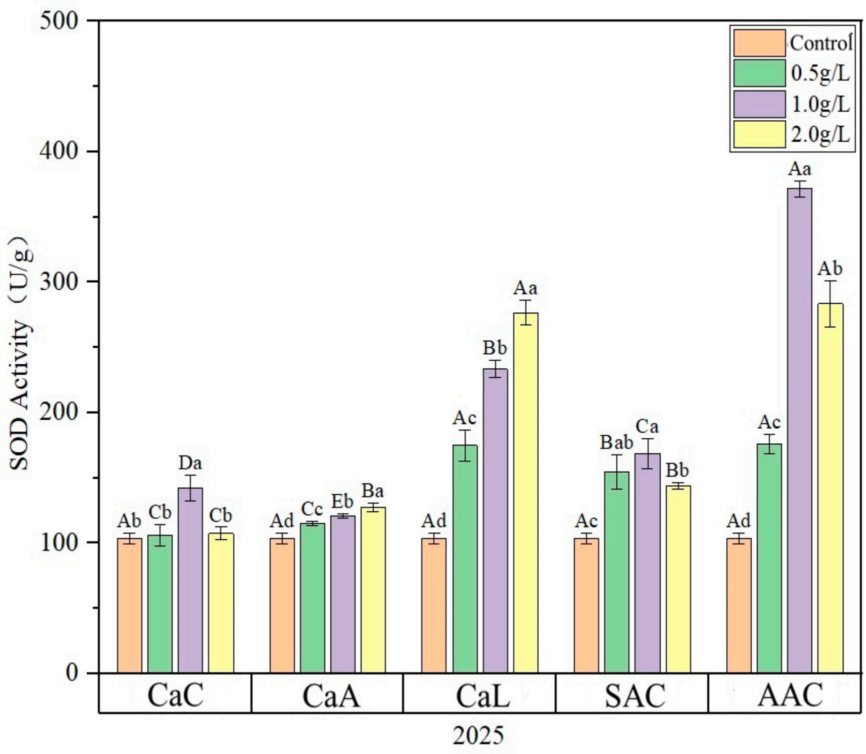

Figure S1. Effects of spraying different forms of calcium fertilizers on the SOD activity of blue honeysuckle fruit in 2025. In the figure, the control represents spraying with water; CaC represents calcium chloride; CaA represents calcium acetate; CaL represents calcium lactate; SAC represents sugar alcohol calcium; AAC represents amino acid calcium. Data are presented as mean ± standard deviation. Different lowercase letters indicate significant differences among different concentrations of the same calcium fertilizer treatment; different uppercase letters indicate significant differences among different calcium fertilizer treatments at the same concentration ($P < 0.05$).

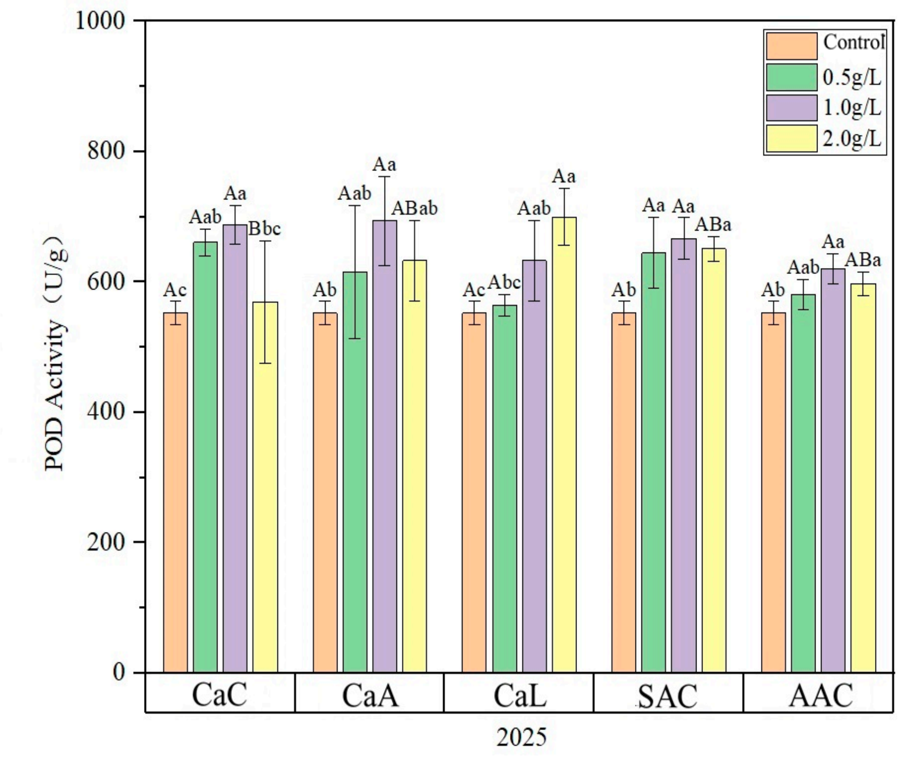

Figure S2. Effects of spraying different forms of calcium fertilizers on the POD activity of blue honeysuckle fruit in 2025. In the figure, the control represents spraying with water; CaC represents calcium chloride; CaA represents calcium acetate; CaL represents calcium lactate; SAC represents sugar alcohol calcium; AAC represents amino acid calcium. Data are presented as mean ± standard deviation. Different lowercase letters indicate significant differences among different concentrations of the same calcium fertilizer treatment; different uppercase letters indicate significant differences among different calcium fertilizer treatments at the same concentration ($P < 0.05$).

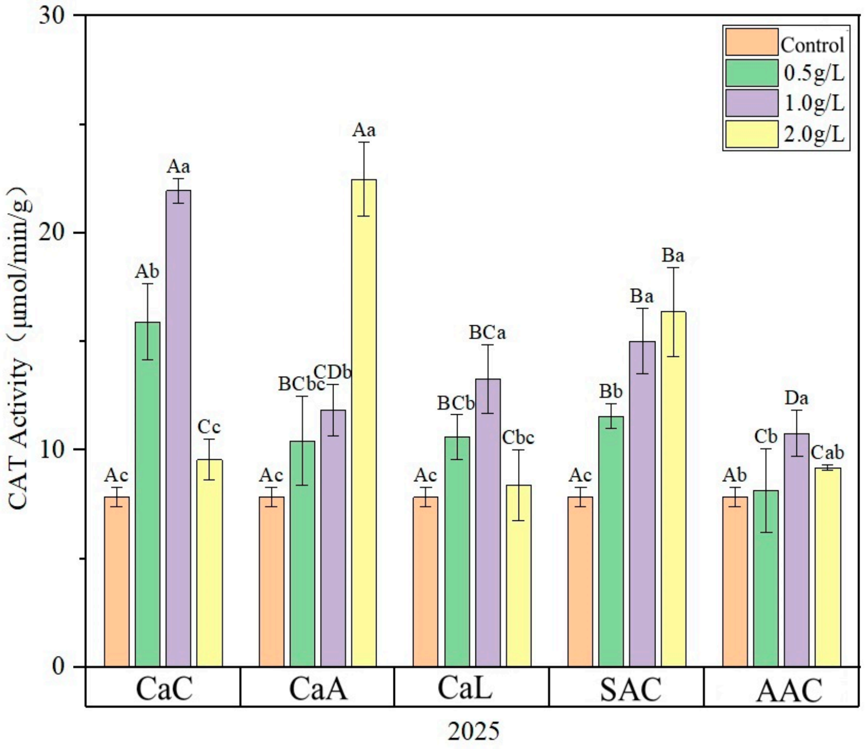

Figure S3. Effects of spraying different forms of calcium fertilizers on the CAT activity of blue honeysuckle fruit in 2025. In the figure, the control represents spraying with water; CaC represents calcium chloride; CaA represents calcium acetate; CaL represents calcium lactate; SAC represents sugar alcohol calcium; AAC represents amino acid calcium. Data are presented as mean ± standard deviation. Different lowercase letters indicate significant differences among different concentrations of the same calcium fertilizer treatment; different uppercase letters indicate significant differences among different calcium fertilizer treatments at the same concentration ($P < 0.05$).

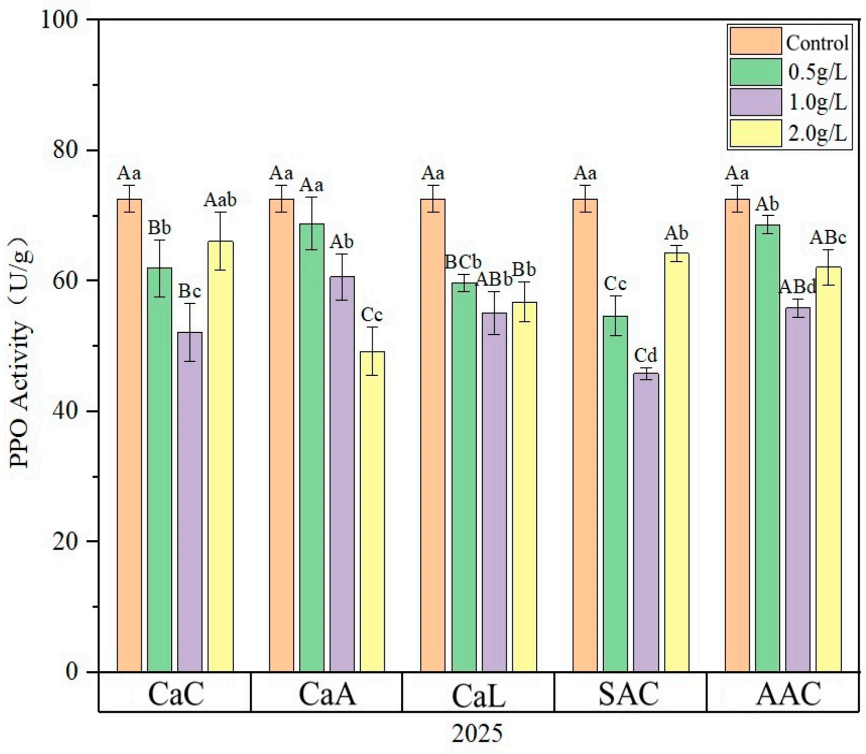

Figure S4. Effects of spraying different forms of calcium fertilizers on the PPO activity of blue honeysuckle fruit in 2025. In the figure, the control represents spraying with water; CaC represents calcium chloride; CaA represents calcium acetate; CaL represents calcium lactate; SAC represents sugar alcohol calcium; AAC represents amino acid calcium. Data are presented as mean ± standard deviation. Different lowercase letters indicate significant differences among different concentrations of the same calcium fertilizer treatment; different uppercase letters indicate significant differences among different calcium fertilizer treatments at the same concentration ($P < 0.05$).

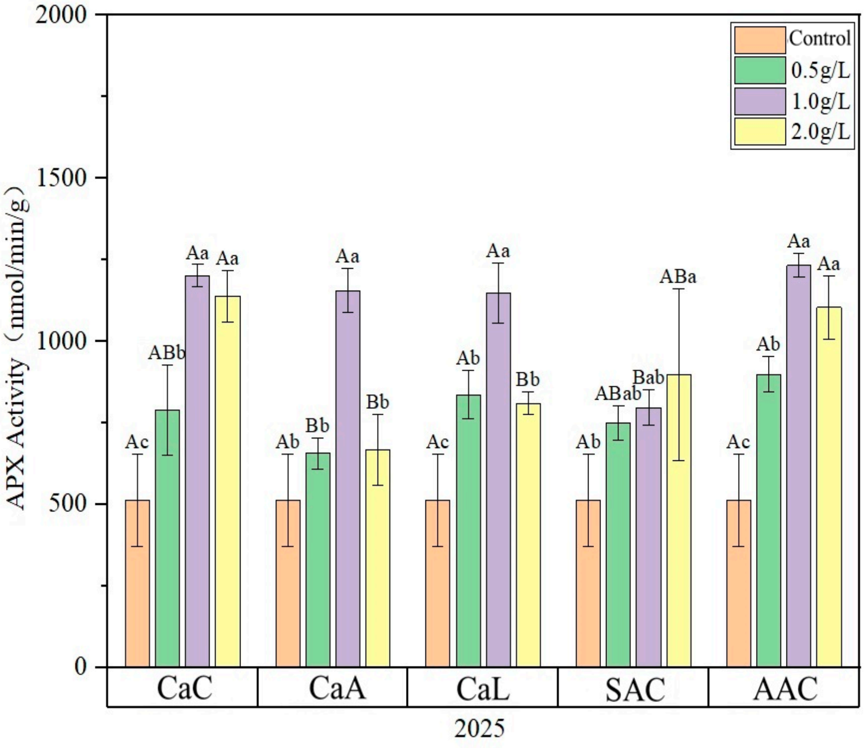

Figure S5. Effects of spraying different forms of calcium fertilizers on the APX activity of blue honeysuckle fruit in 2025. In the figure, the control represents spraying with water; CaC represents calcium chloride; CaA represents calcium acetate; CaL represents calcium lactate; SAC represents sugar alcohol calcium; AAC represents amino acid calcium. Data are presented as mean ± standard deviation. Different lowercase letters indicate significant differences among different concentrations of the same calcium fertilizer treatment; different uppercase letters indicate significant differences among different calcium fertilizer treatments at the same concentration ($P < 0.05$).

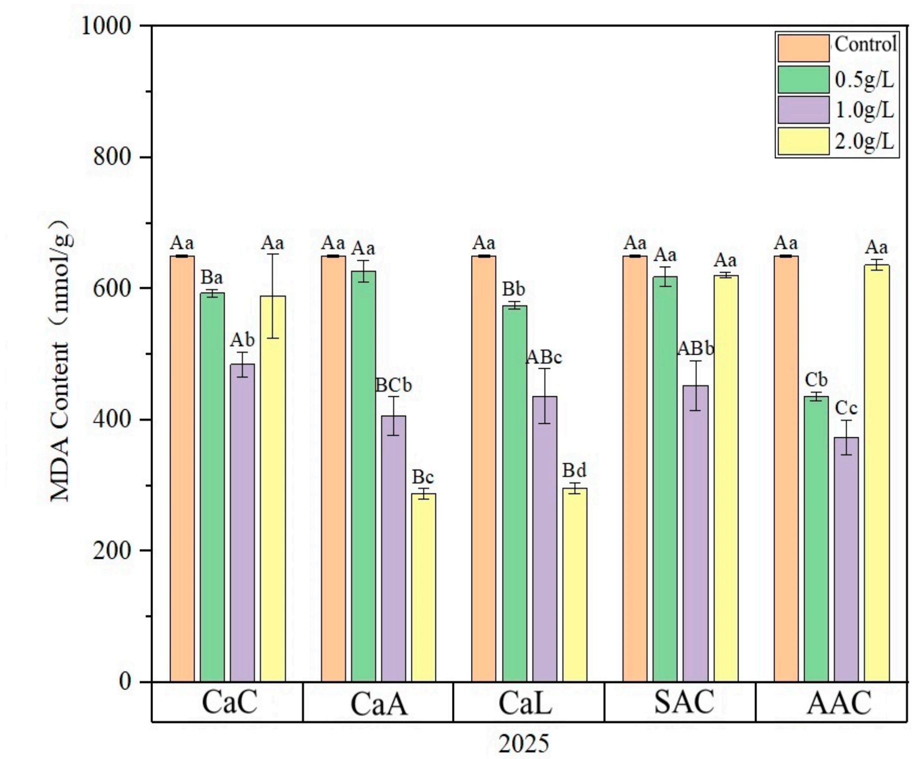

Figure S6. Effects of spraying different forms of calcium fertilizers on the MDA content of blue honeysuckle fruit in 2025. In the figure, the control represents spraying with water; CaC represents calcium chloride; CaA represents calcium acetate; CaL represents calcium lactate; SAC represents sugar alcohol calcium; AAC represents amino acid calcium. Data are presented as mean ± standard deviation. Different lowercase letters indicate significant differences among different concentrations of the same calcium fertilizer treatment; different uppercase letters indicate significant differences among different calcium fertilizer treatments at the same concentration ($P < 0.05$).

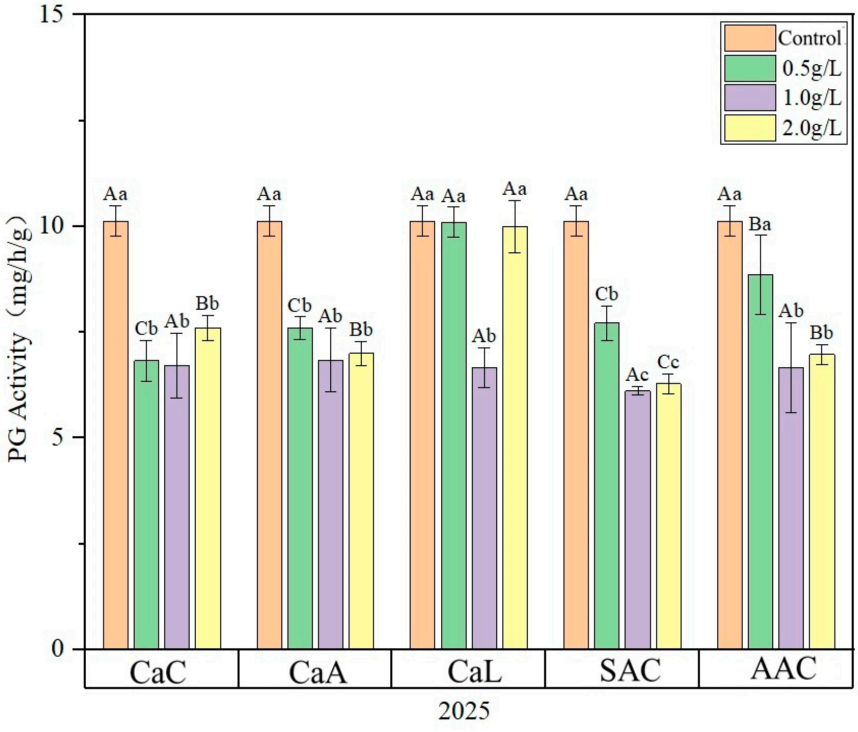

Figure S7. Effects of spraying different forms of calcium fertilizers on the PG activity of blue honeysuckle fruit in 2025. In the figure, the control represents spraying with water; CaC represents calcium chloride; CaA represents calcium acetate; CaL represents calcium lactate; SAC represents sugar alcohol calcium; AAC represents amino acid calcium. Data are presented as mean ± standard deviation. Different lowercase letters indicate significant differences among different concentrations of the same calcium fertilizer treatment; different uppercase letters indicate significant differences among different calcium fertilizer treatments at the same concentration ($P < 0.05$).

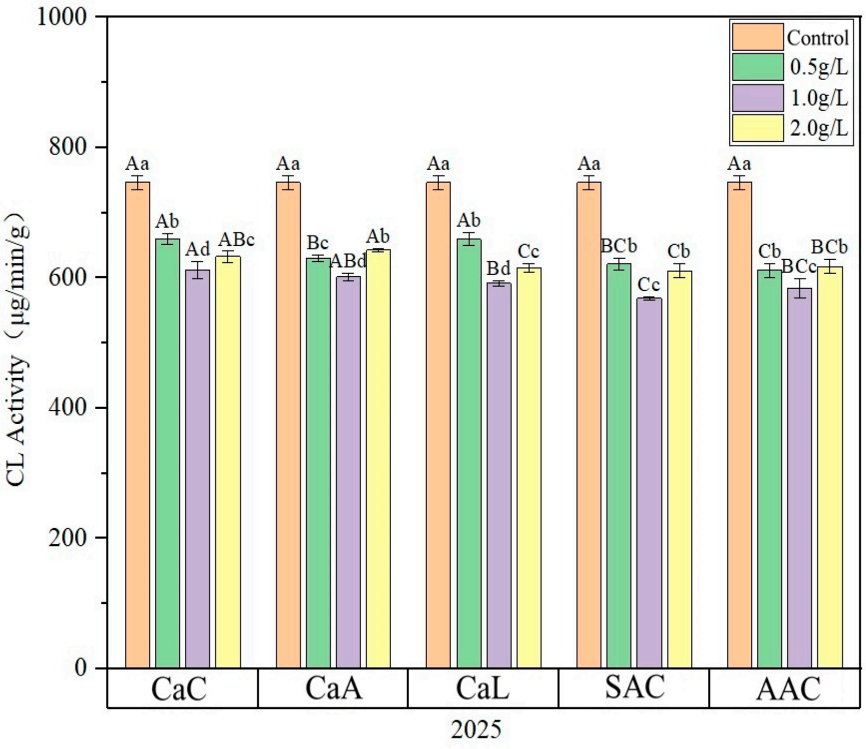

Figure S8. Effects of spraying different forms of calcium fertilizers on the CL activity of blue honeysuckle fruit in 2025. In the figure, the control represents spraying with water; CaC represents calcium chloride; CaA represents calcium acetate; CaL represents calcium lactate; SAC represents sugar alcohol calcium; AAC represents amino acid calcium. Data are presented as mean ± standard deviation. Different lowercase letters indicate significant differences among different concentrations of the same calcium fertilizer treatment; different uppercase letters indicate significant differences among different calcium fertilizer treatments at the same concentration ($P < 0.05$).

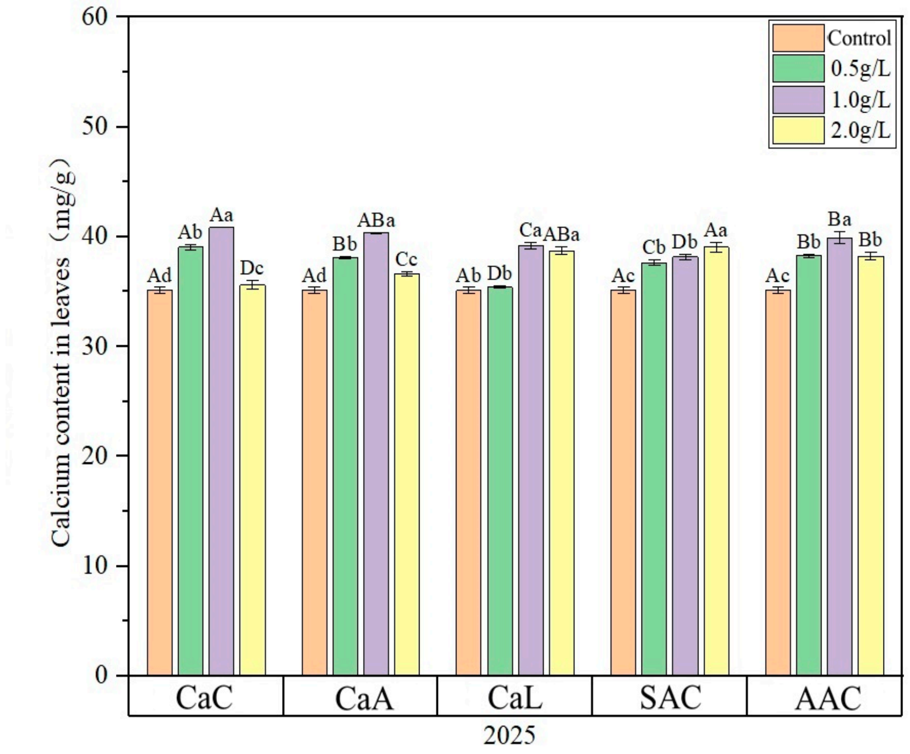

Figure S9. Effects of spraying different forms of calcium fertilizers on the calcium content in blue honeysuckle leaves in 2025. In the figure, the control represents spraying with water; CaC represents calcium chloride; CaA represents calcium acetate; CaL represents calcium lactate; SAC represents sugar alcohol calcium; AAC represents amino acid calcium. Data are presented as mean ± standard deviation. Different lowercase letters indicate significant differences among different concentrations of the same calcium fertilizer treatment; different uppercase letters indicate significant differences among different calcium fertilizer treatments at the same concentration ($P < 0.05$).

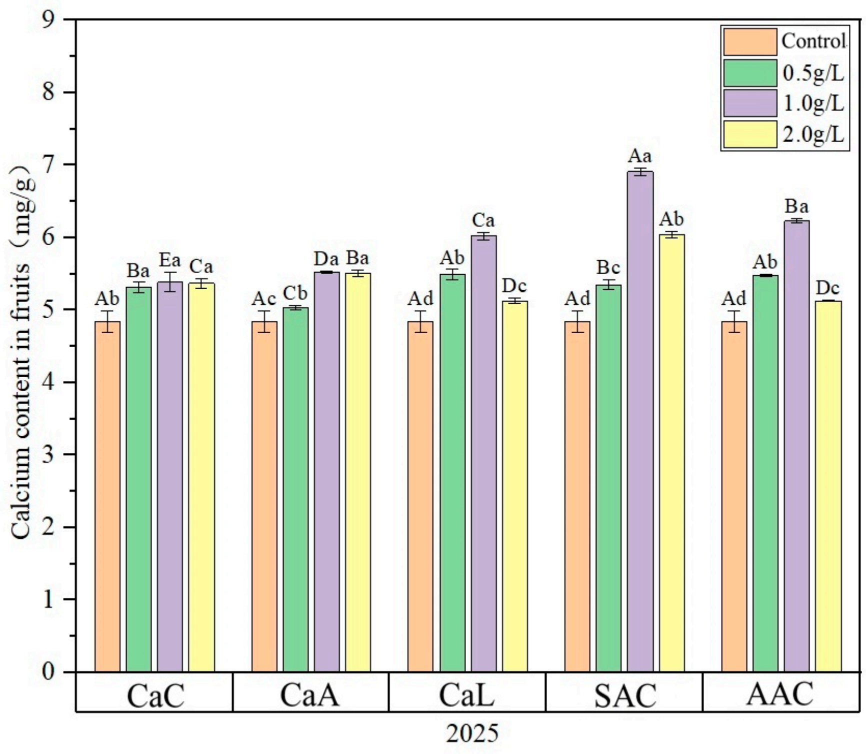

Figure S10. Effects of spraying different forms of calcium fertilizers on the calcium content in blue honeysuckle fruits in 2025. In the figure, the control represents spraying with water; CaC represents calcium chloride; CaA represents calcium acetate; CaL represents calcium lactate; SAC represents sugar alcohol calcium; AAC represents amino acid calcium. Data are presented as mean ± standard deviation. Different lowercase letters indicate significant differences among different concentrations of the same calcium fertilizer treatment; different uppercase letters indicate significant differences among different calcium fertilizer treatments at the same concentration ($P < 0.05$).

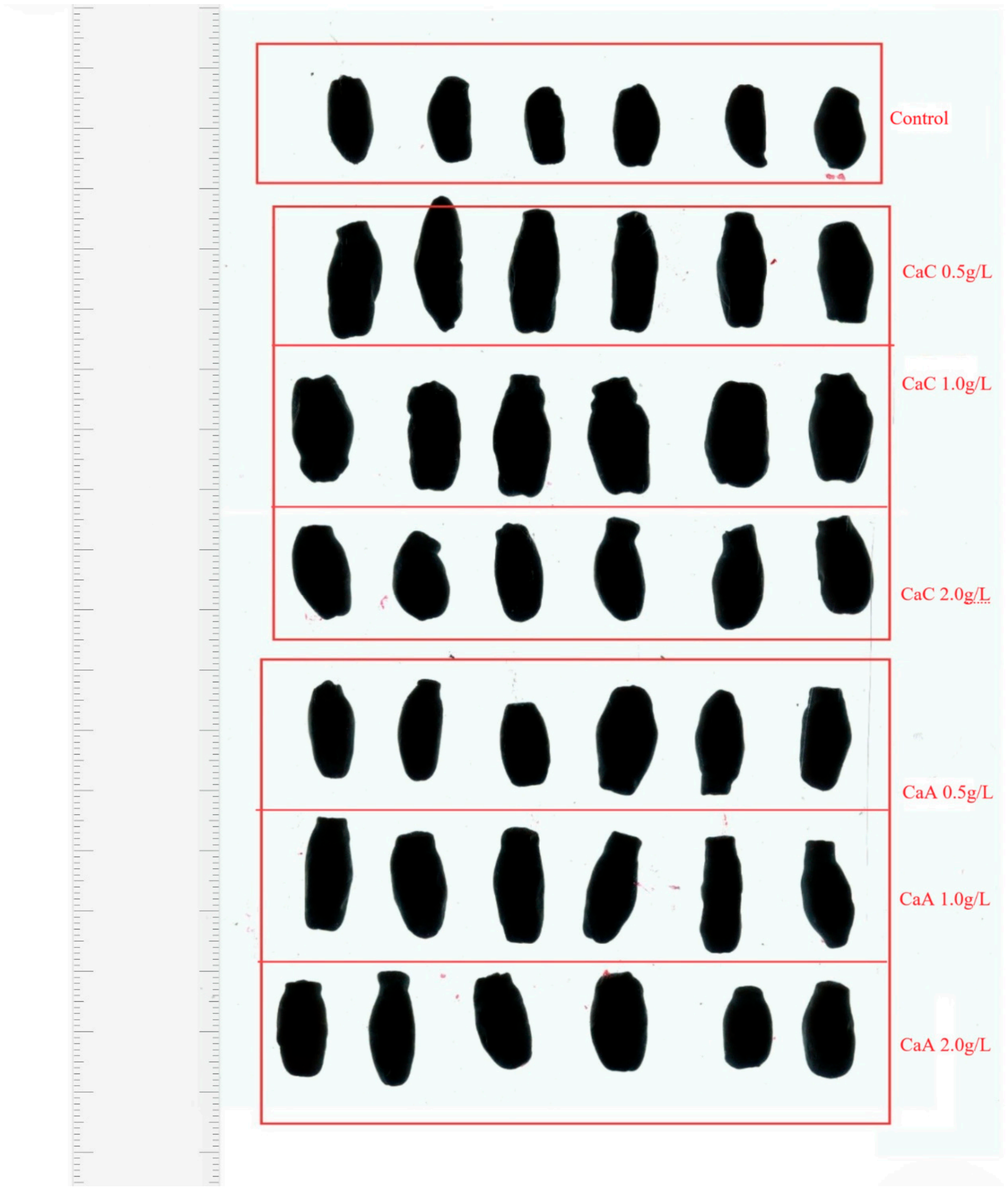

Figure S11. Blue honeysuckle shape diagram. In the figure, the control represents spraying with water; CaC represents calcium chloride; CaA represents calcium acetate. Fruit samples were scanned using an Epson Expression 12000XL scanner and analyzed with WinRHIZO Pro 2005 software (Regent Instruments Inc., Quebec, Canada).

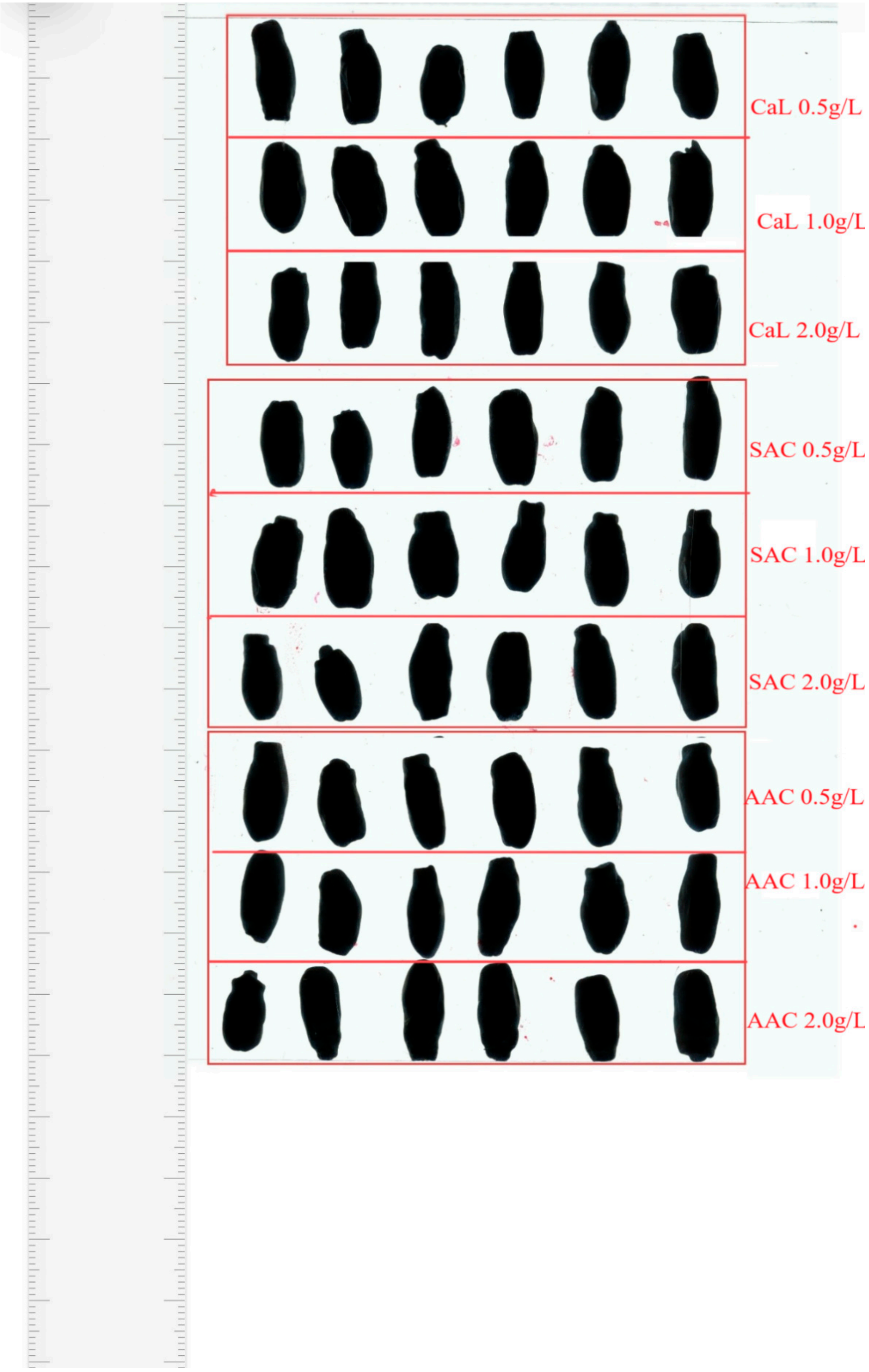

Figure S12. Blue honeysuckle shape diagram (continued). In the figure, the control represents spraying with water; CaC represents calcium chloride; CaA represents calcium acetate. Fruit samples were scanned using an Epson Expression 12000XL scanner and analyzed with WinRHIZO Pro 2005 software (Regent Instruments Inc., Quebec, Canada).
